# Low incidence of COVID-19 case severity and mortality in Africa; Could malaria co-infection provide the missing link?

**DOI:** 10.1186/s12879-022-07064-4

**Published:** 2022-01-22

**Authors:** Silas Acheampong Osei, Robert Peter Biney, Alberta Serwah Anning, Lydia Nkuah Nortey, George Ghartey-Kwansah

**Affiliations:** 1 School of Pharmacy and Pharmaceutical Sciences, University of Cape Coast, Cape Coast, Ghana; 2 Department of Biomedical Sciences, University of Cape Coast, Cape Coast, Ghana

**Keywords:** COVID-19, Malaria, *Plasmodium falciparum*, SARS-CoV-2, Malaria-COVID 19 co-infection

## Abstract

**Background:**

Despite reports of malaria and coronavirus diseases 2019 (COVID-19) co-infection, malaria-endemic regions have so far recorded fewer cases of COVID-19 and deaths from COVID-19, indicating a probable protection from the poor outcome of COVID-19 by malaria. On the contrary, other evidence suggests that malaria might contribute to the death caused by COVID-19. Hence, this paper reviewed existing evidence hypothesizing poor outcome or protection of COVID-19 patients when co-infected with malaria.

**Methods:**

PRISMA guidelines for systematic review were employed in this study. Published articles from December 2019 to May 2021on COVID-19 and malaria co-infection and outcome were systematically searched in relevant and accessible databases following a pre-defined strategy. Studies involving human, in vivo animal studies, and in vitro studies were included.

**Results:**

Twenty three (23) studies were included in the review out of the 3866 records identified in the selected scientific databases. Nine (9) papers reported on co-infection of COVID-19 and malaria. Five (5) papers provided information about synergism of malaria and COVID-19 poor prognosis, 2 papers reported on syndemic of COVID-19 and malaria intervention, and 7 studies indicated that malaria protects individuals from COVID-19.

**Conclusions:**

Low incidence of COVID-19 in malaria-endemic regions supports the hypothesis that COVID-19 poor prognosis is prevented by malaria. Although further studies are required to ascertain this hypothesis, cross-immunity and common immunodominant isotopes provide strong evidence to support this hypothesis. Also, increase in co-inhibitory receptors and atypical memory B cells indicate synergy between COVID-19 and malaria outcome, though, more studies are required to make a definite conclusion.

## Highlights

- Low incidence and mortality rate of COVID-19 in malaria endemic regions
- There have been reports of malaria and COVID-19 co-infection
- Malaria could be the link for the reported low incidence of COVID-19 in Africa
- Cross immunity and common immunodominant epitopes between Malaria and COVID-19, and antimalaria drugs could be the reason for observed low fatality and incidence rate of COVID-19 in malaria endemic regions.
- Malaria infection and deaths could increase amid the COVID-19 pandemic due to interrupted malaria control interventions.

## Introduction

Malaria is an infectious disease caused by unicellular protozoans from the *Plasmodium* genus. Five species of *Plasmodium*; *falciparum*, *ovale*, *vivax*, *malariae,* and *knowlesi*, are known to infect humans [1, 2]. Malaria is still a major global health burden. Several strategies put in place to eradicate malaria has proven futile, especially in the sub-Saharan Africa region which still records about 230 million cases annually. The World Health Organization (WHO) estimates that 40% of the world's population is at risk of malaria infection, making it a major global infectious disease [3].

Corona Virus Disease of 2019 (COVID-19) however, is a novel coronavirus-causing acute respiratory disease. It was first reported in December 2019 in Wuhan, China. Cases skyrocketed within a few weeks causing a global epidemic by March 11, 2020 [4, 5]. The disease which is caused by the severe acute respiratory syndrome coronavirus 2 (SARS-CoV-2), unprecedently caused a global pandemic to date, and is responsible for about 4.3 million deaths and counting [6]. There are similarities between symptoms of malaria and COVID-19, especially in their initial symptomatology. For example, symptoms such as fever, acute onset headache, tiredness, are marked in both COVID-19 and malaria. The common symptoms usually lead to misdiagnosis of malaria for COVID-19 and vice versa especially in the early stages of the disease when symptoms are mild and quiet undefined [7].

Malaria and COVID-19 co-infection has been reported in both malaria-endemic [8, 9] and non-endemic regions [9, 10]. These co-infections can be devastating especially in pregnant women and children, who have always been the most vulnerable group in malaria-associated death [11]. The outcome of malaria-COVID-19 co-infection can be fatal, due to the overlapping symptoms which might delay diagnosis. Also, the suspension of various malaria intervention programs in some malaria-endemic regions during the COVID-19 pandemic to refocus resources on the deadly pandemic has been known to contribute to an increase in the number of malaria cases and death [12].

On the contrary, there have been reports that suggests that malaria may confer protection against COVID-19 due to the low prevalence of COVID-19 in endemic malaria regions [13, 14]. Indeed, Africa, the most endemic malaria region, as of September 2021, recorded only 5.5 million cases, representing approximately 2.6% of the 218.2 million global confirmed cases [6]. Mortality statistics also indicates that Africa records 146.39 deaths per million population whilst Europe and the United States records1,590.98 and 1,960.43 deaths per million population, respectively [15, 16]. Thus, the increased prevalence and death rate of COVID-19 in Americans, especially African Americans [17, 18], dispute the assumption that SARS-CoV-2 is not virulent in black people. Hence malaria has been attributed to the low incidence and mortality of COVID-19 in the endemic regions.

Against the background of these contrasting reports, this review seeks to summarize the pieces of evidence suggesting that a co-infection of malaria and COVID-19 either leads to poor clinical outcomes or vice versa using scientific data published since December 2019 to May 2021.

## Methods

This systematic review follows the guideline of PRISMA (Preferred Reporting Items for Systematic Reviews and Meta-Analyses [19].

### Study inclusion and exclusion criteria

All accessible and available data published from December 2019 to May 2021 on the COVID-19 and malaria was considered. In vitro, in vivo animals and human studies on COVID-19 and malaria co-infection were included. Report from post-mortem findings on COVID-19 and malaria co-infection were also included. However, studies on drug treatment for COVID-19 and malaria co-morbidity were excluded.

### Search strategy and selection criteria

The search was performed in recognized electronic databases for peer-reviewed papers on the subject. Databases including Google Scholar, ResearchGate, PubMed, CINAHL and Medline Plus were searched for studies published in any language. Search was performed by entering the following keywords (“Malaria and COVID-19, and Malaria and COVID-19 coinfection”) and included a broad derivative for wide search. We also retrieved articles with relevant and accessible abstracts with an unclear title. Other articles were manually searched, and references identified, and when appropriate, included in the review.

### Data extraction

The identified electronic reports were imported into *Endnote* references manager which was also used to remove duplicates. The abstract and title of each paper were screened, followed by a full-text review to make sure the study met the inclusion criteria. Two reviewers, SAO and GGK, did the first assessment. The second assessment was done by RPB, ASA, and LNN.

Predetermined study objectives were defined for extraction and documentation by GGK and SAO. The results were then grouped into study types.

## Results

### Study selection

**Fig. 1:**
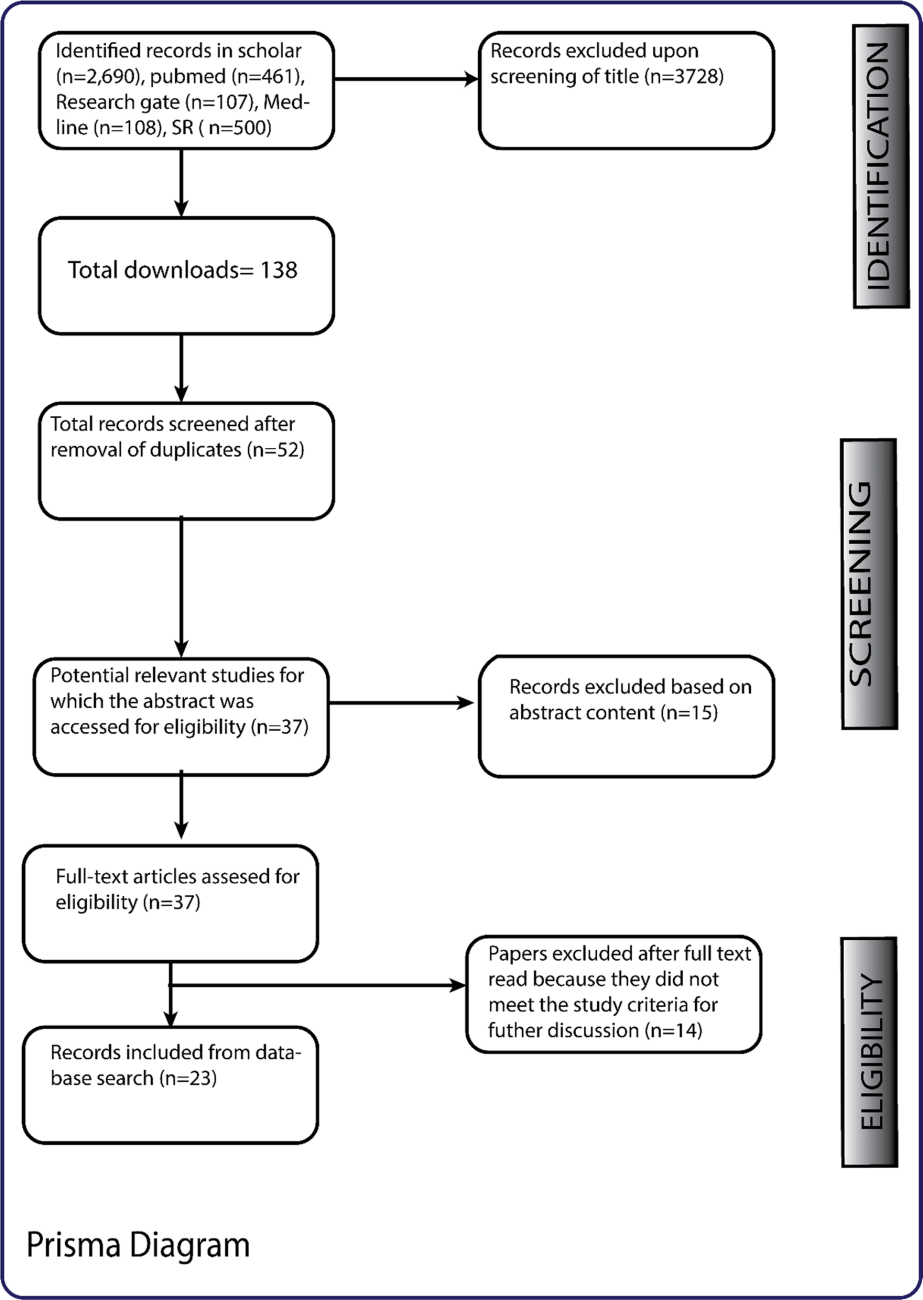
The flowchart of study identification and study selection process

3866 records were identified from all the electronic databases which were included in the search. From this list, 138 papers were downloaded after review based on their titles and accessibility. Out of the total downloads, we identified 52 relevant literatures after duplicates were removed. All the 52 papers were selected for full-text assessment, and of these, 29 papers were excluded leaving 23 papers reporting on 4 topics for further extraction (Fig. 1).

Papers were excluded if [1] it was a review, news, comments, or a letter to an editor (n = 57); [2] it was case report or description of COVID-19 and malaria co-infection without further study (n = 11), was on the impact of COVID-19 on malaria control interventions (n = 10) or on malaria drugs used in the treatment of COVID-19 [8] (Fig. 1).

### Study characteristics and design

**Fig. 2:**
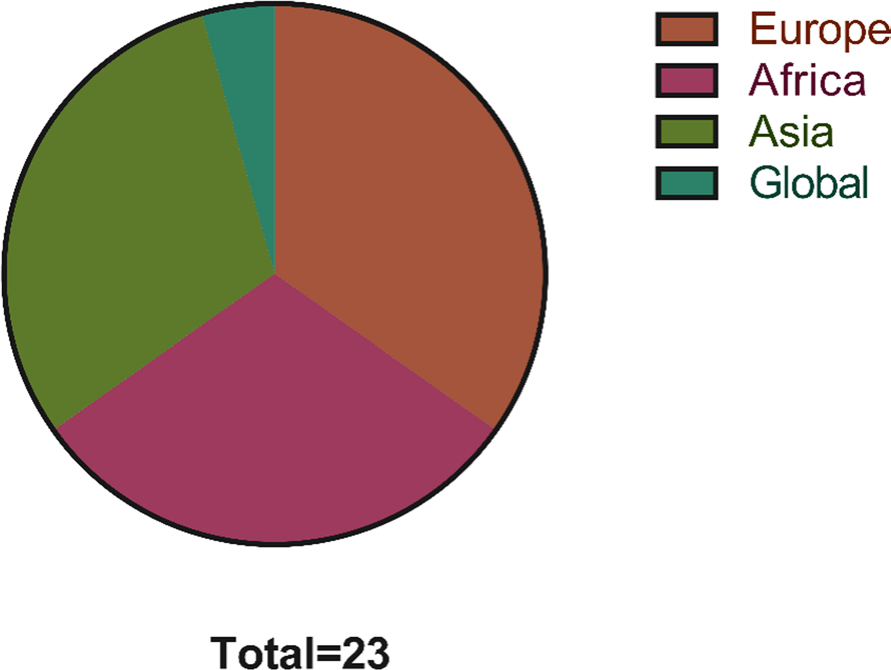
A pie chart showing the characteristics of included studies. 8 papers were conducted in Europe, 7 were obtained from Asia, 7 were extracted from studies conducted in Africa, and 1 study involved collaboration from different continents (global)

Table 1 summarizes all the papers included in the study. Study was taken from global findings on the subject which include reports from Africa (n = 7), Europe (n = 8), Asia (n = 7), and global (n = 1) (Fig. 2). Nine studies reported on the co-infection of malaria and COVID-19, 5 articles studied the poor outcome of COVID-19 and malaria co-infection, 7 studies described the contra-synergic interaction between malaria and COVID-19, and 2 studies were about the impact of malaria on COVID-19 mortality (Table 1).

**Table 1:** Summary of included studies and their sources

Study type	Number of studies	References
Co-infection reports	9	[8–10], [20–25]
COVID-19 and malaria synergy	5	[26–30]
Malaria protection against COVID-19	7	[13, 14], [31–35]
Syndemic impacts of COVID-19 on malaria	2	[36, 37]

## Discussion

### Malaria and COVID-19 co-infection

The similar clinical presentations of malaria and COVID-19 makes it difficult to distinguish between these diseases, especially in endemic areas [10, 38, 39]. This poses a challenge for differential diagnosis of COVID-19 and malaria. The first reported case of COVID-19 and malaria co-infection was in India, in which a 55-year-old man was diagnosed with SARS-CoV-2 and associated imported *Plasmodium vivax* [23]. Mahajan and colleagues also reported co-infection of malaria, COVID-19, and dengue fever mostly in low-and middle-income countries (LMICs) [9]. Indeed, LMICs face the danger of COVID-19 co-infection with other infectious diseases. Since malaria is more predominant in Sub-Saharan Africa and South-East Asia, most of this co-infection is recorded in these regions. Another report from Mahajan et al., 2020, indicates that not only pregnant women in LMICs are prone to malaria and COVID-19 co-infection, health care workers (HCWs) are also at a higher risk of co-infection. Probably due to the constant exposure to COVID-19 patients and late diagnosis of the disease as a result of the overlapping symptoms between malaria and COVID-19. The group conducted a retrospective study in a COVID-19 dedicated hospital in India and realized that out of the 3711 HCWs in the hospital, 491 had COVID-19 and 31 were co-infected with malaria [9]. Malaria-COVID-19 co-infection has since then not limited to one region. It was not surprising that Onosakponome and Wogu reported co-infection of malaria and COVID-19 in Nigeria, a suburb of Sub-Saharan Africa because although reports of COVID-19 in this region is relatively low, malaria is very prominent. However there have also been reports of malaria and COVID-19 co-morbidity in Europe [8] where malaria is not endemic. Incidence of co-infection in non-endemic areas could be deleterious due to lack of immunity and misdiagnosis since malaria and COVID-19 symptoms overlap.

### Covid-19 surveillance in malaria endemic countries

A working disease surveillance system is critical for rapid establishment of intervention measures during an outbreak. In cases of unprecedented global health threat such as the COVID-19 pandemic, it helps to priorities allocation of resources, preparedness, monitoring and evaluation of intervention programs [40]. Previous records indicate a poor disease surveillance system in Africa. We can recall that Ebola virus disease (EVD) went unnoticed for months as a result of poor surveillance system which delayed intervention measures and led to increased death rate of infected individuals [41]. Recent studies have also identified a gap in epidemiologic data collection and its implementation to address health menace at national levels in Africa [42].

The weak disease surveillance system in Africa is worsened at the emergence of COVID-19 pandemic as it has hampered treatment, testing, immunization and surveillance  efforts for other infectious diseases such as malaria [43–45]. Several countries in Africa have switched focus towards COVID-19 care which results in ambiguous surveillance data for other infectious diseases. Some countries like Egypt has adopted measures to improve their surveillance system to track COVID-19 disease, improved their healthcare system and most importantly, established a national Egyptian Public Health system to enable health authorities to monitor the extent of the pandemic in Egypt [46] whilst countries like Burundi and Congo lack sufficient interventions with regards to COVID-19 surveillance as a result of inadequate logistics and failure to learn from previous Ebola outbreak [47].

The poor   disease surveillance system in malaria endemic countries, such as in the case of Africa, might account for the observed low number of COVID-19 cases. For instance, Nigeria has a large population of over 200 million people, a study conducted by Tunde and colleagues indicate that only 1,997,479 (1%) of the population had tested for COVID-19 as at May 2021 [48]. Thus, the capacity for Africa to rapidly detect and test for COVID-19 cases is hugely undermined by the weak surveillance system.

### Impact of other infectious diseases amid COVID-19 pandemic

Africa has been burdened with several infectious diseases from the past three decades and it poses a significant threat to public health and economic growth on the African continent [49]. Until the onset of COVID-19 pandemic, other infectious diseases like malaria, Dengue fever, tuberculosis, and Ebola were a major problem on the continent. However, since COVID-19 was declared as a pandemic, attention has been shifted to only COVID-19, and has interrupted several intervention programs for equally health threatening infectious diseases like malaria.

Recent reports indicate a rise in other infectious diseases in Africa as resources has been shifted in curbing the negative impact of COVID-19. For instance, eradication of infections like Dengue fever can be prevented with mass vaccination and vector control which needs several resources and intervention programs to implement, but unfortunately, COVID-19 pandemic has interrupted these measures, especially the vaccination campaigns, due to restrictive measures to control the disease [50]. In Democratic Republic of Congo, there was re-emergence of measles outbreak during the COVID-19 pandemic as a result of the country’s inadequate resources overburdened with COVID-19 measures and poor disease surveillance system that was not able to integrate measles report into the country’s disease surveillance system [51].

Lassa fever (LF) is responsible for 5000 to 1000 deaths per year, predominantly in West Africa. The symptoms of Lassa fever, such as sore throat, chest pains, pharynx respiratory symptoms, loss of hearing and hemorrhagic fever, overlaps with COVID-19 symptoms. This accounts for misdiagnosis and late detection of LF which progress to the later stage of the disease. A significant increase in LF cases was observed from 2019 to early 2020 with 3.5% increase  of intensity febrile sick children and resulted in 24%-33% fatality even with ribavirin treatment [43].

In February 2021, Democratic Republic of Congo reported a new EBV outbreak amid the COVID-19 pandemic. Although the outbreak was successfully curtailed, it led to a rise    in the number of COVID-19 cases [52]. This indicates that the health system in DRC, like most African countries, is incapable of managing co-existence of both outbreaks, hence, the neglect of other infectious diseases to manage COVID-19 instead.

In spite of these, the total number of cases and fatality rate of COVID-19 in Africa is relatively low as compared to Europe and America who are not faced with the above discussed infectious diseases. The common infectious disease that is prevalent on the African continent and other LMICs is malaria, and among these countries the fatality rate of COVID-19, as until today, remains low. Thus, there is a possibility that malaria is conferring protection in individuals co-infected with malaria and COVID-19.

### COVID-19 and malaria inverse relationship

#### Low incidence of COVID-19 in malaria-endemic regions

An epidemiological paradox study in the early stages of COVID-19 pandemic indicated that malaria-endemic regions had low prevalence of COVID-19 [31]. This is surprising since the World Health Organization (WHO) and scholars predicted a huge negative impact of the pandemic on LMICs due to scarce economic resources, poor health systems, and the endemic presence of other infectious diseases such as Ebola hemorrhagic fever which might weaken the immune system [53]. Even in countries where malaria is not endemic, COVID-19 cases have been reported to be low in regions where malaria incidence is high. A study in Italy has reported that the lowest number of COVID-19 cases are seen in areas with the highest malaria incidence [32].Although the inadequate diagnostic techniques and equipment for accurate diagnosis of COVID-19 infection and mortality could account for the low number of pneumonia associated deaths, the hypothesis derived from epidemiological data shows a disproportionate spread of COVID-19 in malaria endemic regions. The inverse relationship between malaria and other coronavirus was reported even before COVID-19 incidence; the distribution of previous coronavirus diseases such Middle East Respiratory Syndrome Coronavirus (MERS-CoV) and SARS indicated that places with high *Plasmodium* parasite infection records low incidences of these diseases [54].

#### Antimalaria drugs inhibit SARS-CoV-2 replication

The use of antimalaria drugs might also account for the observed low mortality rate of SARS-CoV-2 infection in malaria-endemic areas. Studies indicate that certain routinely-used malaria drugs mostly have anti-viral activity; artemisinin derivatives showed activity against cytomegalovirus [13, 55], amodiaquine was active in vitro against SARS-CoV-1 [56], mefloquine exerted in vitro cytopathic effects on SARS-CoV-2 vero cells at 10 μM [57] and pyronaridine administration inhibited Ebola virus replication in vitro with an EC_50_ of 1.14 μM after an hour of infection [58]. A recent study by Gendrot and colleagues also revealed that chloroquine, hydroxychloroquine, ferroquine, desethylamodiaquine, mefloquine, pyronaridine, and quinine inhibited SARS-CoV-2 viral growth in vitro in a standard antimalarial drug regimen [13]. Interestingly, the activities shown for these antimalarial drugs was higher than lopinavir, remdesivir or ritonavir which are drugs being evaluated in clinical trials. In malaria-endemic regions, most individuals are routinely exposed to these agents and it is hypothesized that, this could contribute to the lower incidence of COVID-19 in these regions.

The hypothesis that the use of antimalarials might contribute to low mortality rates in malaria-endemic regions is hugely supported by Ahmad and colleagues report [31]. By employing pharmacoinformatics, Ahmad et al. docked malaria-box compounds (MB) against SARS-CoV-2 main protein (M^pro^) and discovered that 3 compounds, MB_241, MB_250 and MB_266, had a higher docking affinity score, than reference molecules. Strikingly, the root mean square deviation (RMSD) and root mean square fluctuation (RMSF) during simulation indicated that these agents have a more stable binding with SARS-CoV-2 M^pro^ [14].

Considering the fact that antimalarial agents can also act as antiviral agents against various viruses including SARS-CoV-2 and the fact that most individuals are exposed to these agents in malaria-endemic regions it is not far-fetched to postulate that, the administration of antimalarial agents in endemic regions prevents COVID-19 poor prognosis. There is also the possibility that, because symptoms of malaria and COVID-19 overlaps, COVID-19 patients might actually have been treated with antimalarials before a definite diagnosis is made.

### Genetic evidence of COVID-19 and malaria inverse relationships

#### Blood groups and ACE2 receptor

The low incidence and death rate in COVID-19 patients in malaria-endemic regions can be clarified further by the recent reports of shared immunodominant epitopes between *P. falciparum* and SARS-CoV-2. Genomic analyses which filtered the angiotensin-converting enzyme 2 (ACE2) and malaria associated-variants identified 6 candidate genes that are likely predictors for major resistance (or susceptibility) to malaria and higher/lower COVID-19 incidence and severity. These genetic variants included blood group O and reduced ACE2 receptor expression [32].

Indeed, a low incidence of COVID-19 has been reported in blood group O individuals [32]. Zietz and colleagues sought to understand the association between blood groups and COVID-19 risks of infection, intubation and death. Strikingly, they discovered, after adjusting for ancestry by proxies of race and ethnicity, individuals with blood group O had a lower risk of infection [59]. Similar findings were also reported in an earlier study for SARS-CoV-1 and SARS patients [60]. In malaria, blood group O confer resistance to the parasite via reducing parasite rosetting, a mechanism crucial for parasite survival in the host. Rowe and colleagues investigated the impact of erythrocyte polymorphism on malaria pathogenesis in 567 Malawian children and observed that only 21% of children with blood group O developed severe malaria. Also, the odds for an individual with blood group O to develop severe malaria was reduced to 66% compared to other blood groups. Further studies revealed that a reduction in *P. falciparum* rosetting in the parasite isolates from blood group O children [61].

ACE2 receptor has been reported to be the main receptor for cellular entry of SARS-CoV-2 into the human body [62, 63]. Earlier studies indicate that angiotensin II (ANG II) suppresses the development of *Plasmodium gallinaceum* in birds. ANG II is capable of suppressing sporozoites growth in the salivary gland of mosquitoes via a direct disturbance of the parasite membrane [64]. The hypothesis that ANG II confer protection has been documented in other studies [65–67]. The deletion/insertion polymorphism in intron 16 distinguishes ACE1 enzyme. The presence of this polymorphism is associated to the alteration of either bound-forms or circulatory ACE. In cases when D allele dominates, this is linked to a reduction in ACE2 receptor expression and might confer protection against COVID-19. A genetic association study revealed that when D-allele ACE1/D polymorphism is present, the production of ANG II also increases and it often associated with mild malaria. The distribution of D-allele varies across the globe [68]. Africans have been demonstrated to have increased plasma levels of ANG II leading to ACE1/D polymorphism. Delanghe and colleagues [69] supported this when they reported that the log-transformed prevalence of SARS-CoV-2 infection was seen indirectly related to the frequency of ACE D allele frequency and almost 38% or the changes in prevalence can be due to relative frequency of the ACE1 D-allele [70].

In summary, populations in endemic regions develop some immunity against *Plasmodium* infections, hence severe malaria is rare among adults in these regions, however, mild malaria is predominant. This might explain the increase in ANG II in such populations and as such the decrease level of ACE2 receptor which subsequently causes the low prevalence of COVID-19 in the region.

#### Common immunodominant epitopes

Immunogenic cross-reactivity resulting from shared immunodominant epitopes between SARS-CoV-2 and *P. falciparum* antigens might be contributing to the low incidence and death rate of COVID-19 in malaria-endemic areas. There has been reports of the conservation of the apparent immunodominant epitope, thrombospondin-related anonymous protein (TRAP), between *P. falciparum* which causes malaria and SARS-CoV-2 which causes COVID-19 [35]. This shared epitope lies within antigens that help *P. falciparum* invade erythrocytes during infection and may be an alternative route for SARS-CoV-2 entry through the erythrocyte CD127 receptor. Iesa and colleagues [35] searched T-cell-immunodominant epitopes and T-cell major histocompatibility complex (MHC)-restricted epitopes for sequences shared between COVID-19 virus and *P. falciparum* and found several shared tetrapeptides and pentapeptides epitopes [35] including N protein from SARS-CoV/SARS-CoV-2 and *P. falciparum* TRAP, S protein–SARS-CoV-2/SARS-CoV and the predicted epitope in SSP-2 from *P. falciparum*. Both epitopes are capable of stimulating CD8^+^ T-lymphocytes response separately via the recognition of HLA-A*02:01.

Furthermore, four amino acid determinants identified in MHCI-restricted CD8 + T-cells epitopes in TRAP are shared by the nucleocapsid protein 219-LALLLLDRL-227 of SARS-CoV-2 which is restricted to the same recognition site, HLA-A*02:01 [71]. It can be assumed that the cellular adaptive immunity developed against 504-GLALLACAGL-513-immunodominant epitope could recognize 219-LALLLLDRL-227-HLA-A*02:01 complexes originating from SARS-CoV-2 infection and initiate immune response which probably contributes to the early recovery of COVID-19 patients in malaria-endemic regions. In summary, immunogenic cross-reactivity resulting from shared immunodominant epitopes between SARS-CoV-2 *P. falciparum* antigens might explain the low incidence and death rate of COVID-19 in malaria-endemic areas when compared to the non-malaria endemic places, although, further research is needed to validate this hypothesis.

#### Cross immunity

Regarding COVID-19, it is now well known that a considerable percentage of adults are not infected even when exposed to the SARS-CoV-2, and several studies suggest preexisting immunity as the possible primary indication. Raham [33] tested this hypothesis by looking at how innate and heterogenous immunity conferred by tuberculosis (TB) and malaria influences COVID-19 incidence and mortality in malaria-endemic countries. Hierarchical multiple regression analyses for the 80 malaria-endemic countries revealed TB as the direct factor and malaria as the intermediate. The results showed that, although, TB prevalence correlated to a reduction in COVID-19 mortality, an additional effect of reducing COVID-19 mortality with high significant association was observed for malaria [33]. Further evidence in support of this is adduced by report that, there is a significant positive association between malaria elimination date and COVID-19 mortality. Countries that had only recently eradicated malaria had low COVID-19 mortality rate, while matched-counterpart countries which have not recorded malaria cases in the last 15 years had high numbers of COVID-19 mortality [34].

### Synergy of COVID-19 and malaria

While there seem to be a lot of evidence that malaria co-infection with COVID-19 is associated with low incidence and mortality of COVID-19 in malaria-endemic regions, few studies have observed potentiation of COVID-19 mortality due to co-infection with malaria. This has been linked with oxidative stress (8-isoprostaglandin F2 alpha), increased frequencies of plasmablasts and atypical memory B cells as well as the occurrence of T-cell co-inhibitory receptors.

Oxidative stress is very evident in poor outcomes for both malaria and COVID-19 via cytokine storm [69]. "Cytokine storm", proposed as one of the pathogenesis of COVID-19, is a pro-inflammatory response to SARS-CoV-2 infection and also reported in malaria. A study by Muhammed and colleagues [26] reported increased in oxidative marker 8-iso-PGF2α in COVID-19 patients. Meanwhile, 8-iso-PGF2α, has been implicated in the oxidative stress involvement of both COVID-19 and malaria. Also, 8-iso-PGF2α levels were increased significantly for patients infected with COVID 19 and malaria compared to only COVID 19 patients and was directly proportional to malaria parasite density [26]. Thus, there is a potential for synergic oxidative stress effect in malaria-COVID-19 co-infected individuals, however, more studies are needed to ascertain this hypothesis.

T cells play a crucial role in the pathogenesis of infectious diseases including clearing virus-infected cells from the body. Reduction in T cells is correlated with SARS-CoV-2 infection severity [72]. It is not clear how T cell exhaustion and effector cell loss affect pathophysiology of acute infections, However, the upregulation of inhibitory receptors in acute infection can be regarded as a characteristic of the overall immune activation to counterbalance excessive immune responses [73]. There have been reports of increased co-inhibitory receptors, T-cell immunoglobulin mucin-3 (TIM-3) and Lymphocyte-activation gene-3 (LAG-3), in COVID-19-malaria patients [27]. The upregulation and sustained expression of co-inhibitory receptors such as LAG-3, TIM-3 and programmed cell death protein-1 (PD1) is an indication of T cell exhaustion and it is usually found in chronic infections and cancer. Previous studies in malaria show that when co-inhibitory receptors are upregulated in response to acute infection, it could lead to detrimental effects and this can be worsened in a patient co-infected with COVID-19 and malaria.

A recent report indicates that there is a significant reduction in atypical memory B cells and plasmablasts in both COVID-19 and malaria patients [28]. Immune B cells have been postulated to be involved in the pathogenesis of several infectious diseases including malaria and human immune deficiency virus (HIV). An increase in memory B cells loss was associated with activation of the immune cells and severity of COVID-19 patients. Similar results were found in malaria patients. Thus, it can be hypothesized that the low expression of the ectonucleotidase CD73 of B cells contributes to the hyperinflammatory pathophysiology of COVID-19 and the poor outcome in malaria patients. However, the specific role of memory B cells in COVID 19-malaria patients is obscure.

In summary, there is inadequate evidences supporting the synergy of COVID-19 and malaria co-infection and most of the current studies are inferred and hypothesized. Thus, further direct studies are required to reveal the poor prognosis of COVID-19 and malaria co-infection.

### Negative Impact of COVID 19 on malaria control (Future perspective)

In the event that malaria might give protection to COVID-19 fatality, it is worth mentioning that untreated malaria could lead to severe health damage. Malaria has been the leading health burden in Africa since 2000 and has received all the attention regarding control interventions and aids. This can be jeopardized if the COVID-19 pandemic affects the key availability of malaria control interventions. Weiss and colleagues used spatiotemporal Bayesian geostatistical models to assess possible effect of malaria incidence and mortality when malaria control methods are disrupted. Upon estimation of 215.2 million cases and 386.4 thousand deaths across African malaria-endemic regions, Weis et al. discovered that the number of deaths in the endemic places would increase when there is reduction in access to effective antimalarial drug treatment which as a result of global attention shifted to COVID-19. The study postulated that malaria deaths could double in the coming years due to COVID-19 related disruptions to malaria control in Africa.

Another modelling study quantified the extent to which disruptions to malaria, HIV, and tuberculosis services due to COVID-19 overburden in low and middle-income countries could lead to additional mortality in over five years [30]. The results indicated that malaria mortality could decrease by 36% compared with if there was no COVID-19 pandemic. Hagan and friends indicated that this could be a consequence of interruption of planned net campaigns and if not curtailed could lead to the same magnitude of the impact of the COVID-19 pandemic for the next five years in places with the burden of malaria. Thus, it is prudent that amidst the fight to eradicate COVID-19 burden on global health, we do not disrupt malaria interventions in low and middle-income countries who are mostly affected by malaria. If not, the outcome can be catastrophic as seen with COVID-19 today since the Plasmodium parasite is capable of developing resistance to current effective drugs.

It is noteworthy that even if stringent non-pharmaceutical interventions are put in place to curtail malaria, transmission is expected to go higher, amidst the pandemic. A study which accessed the syndemic impact of COVID-19 and malaria in Africa reported a potential increased transmission rate for malaria even during malaria interventions since individuals in these endemic areas fear the risk of being diagnosed with COVID-19 because of the overlapping symptoms between COVID-19 and malaria [36]. This suggest that early intervention of COVID-19 is also necessary to effectively reduce the scale of the epidemic and lessen its effect on malaria transmission potential.

## Conclusion

This paper reviews evidence that malaria might have protection against SARS-CoV-2 infection or contribute to the poor outcome of the disease. Various reports reviewed here show that most people in malaria-endemic regions have some protection against COVID-19. Common immunodominant epitopes and cross-immunity between COVID-19 and malaria give strong evidence as to how malaria might protect COVID-19 and malaria co-infected patients. There are however a few reports of COVID-19 and malaria co-infection leading to poor outcomes in some patients. This has been supported by reports of increase in co-inhibitory receptors and atypical memory B cells during co-infection.

## Recommendation

Ultimately, further research is needed, especially in malaria-endemic countries, to understand how malaria protects individuals infected with COVID-19 which might provide more evidence as to how to combat the epidemic.

## Data Availability

The data are available only upon request from the authors.

## Abbreviations

ACE2: Angiotensin converting enzyme-2
ANG II: Angiotensin II
CINAHL: Cumulative index for Nursing and Allied Health literature
COVID-19: Corona virus disease of 2019
HIV: Immune deficiency virus
LAG-3: Lymphocyte-activating gene-3
LMICs: Low and Middle-income countries
MERS-CoV: Middle East respiratory syndrome
MHC: Major histocompatibility complex
PD1: Programmed cell death protein-1
RMSD: Root mean square deviation
RMSF: Root mean square fluctuation
SARS-CoV-2: Severe acute respiratory syndrome coronavirus-2
TB: Tuberculosis
TIM 3: T-cell immunoglobulin mucin-3
TRAP: Thrombospondin -related anonymous protein
WHO: World Health Organization
